# Expanding the genetic spectrum of primary familial brain calcification due to *SLC2OA2* mutations: a case series

**DOI:** 10.1007/s10048-021-00634-9

**Published:** 2021-01-20

**Authors:** Luca Magistrelli, Roberta Croce, Fabiola De Marchi, Chiara Basagni, Miryam Carecchio, Nicola Nasuelli, Roberto Cantello, Federica Invernizzi, Barbara Garavaglia, Cristoforo Comi, Letizia Mazzini, Sandra D’Alfonso, Lucia Corrado

**Affiliations:** 1 Department of Translational Medicine, Section of Neurology, University of Piemonte Orientale and “Maggiore della Carità” University Hospital, Novara, Italy; 2 PhD Program in Clinical and Experimental Medicine and Medical Humanities, University of Insubria, Varese, Italy; 3 Department of Health Sciences, University of Eastern Piedmont, Novara, Italy; 4 ALS Center, Department of Neurology, Azienda Ospedaliero Universitaria Maggiore della Carità, Novara, Italy; 5 Department of Neuroscience, University of Padua, Padua, Italy; 6 SS Trinità Hospital, Borgomanero, ASL Novara, Novara, Italy; 7 Medical Genetics and Neurogenetics Unit, Fondazione IRCCS Istituto Neurologico Carlo Besta, Milan, Italy

**Keywords:** Primary familil brain calcification, *SLC20A2*, Motor neuron

## Abstract

Primary familial brain calcification (PFBC) is a neurological condition characterized by the presence of intracranial calcifications, mainly involving basal ganglia, thalamus, and dentate nuclei. So far, six genes have been linked to this condition: *SLC20A2*, *PDGFRB*, *PDGFB*, and *XPR1* inherited as autosomal-dominant trait, while *MYORG* and *JAM2* present a recessive pattern of inheritance. Patients mainly present with movement disorders, psychiatric disturbances, and cognitive decline or are completely asymptomatic and calcifications may represent an occasional finding. Here we present three variants in *SLC20A2*, two exonic and one intronic, which we found in patients with PFBC associated to three different clinical phenotypes. One variant is novel and two were already described as variants of uncertain significance. We confirm the pathogenicity of these three variants and suggest a broadening of the phenotypic spectrum associated with mutations in *SLC20A2*.

## Introduction

Primary familial brain calcification (PFBC) is a neurological condition characterized by the presence of intracranial calcifications, mainly involving basal ganglia, thalamus, and dentate nuclei. So far, six genes have been linked to this condition: *SLC20A2*, *PDGFRB*, *PDGFB*, and *XPR1* (inherited as autosomal-dominant trait) [1] while *MYORG* and *JAM2* present a recessive pattern of inheritance [2, 3]

Clinically, patients mainly present with movement disorders, psychiatric disturbances, and cognitive decline or are completely asymptomatic and calcifications may represent an occasional finding [4].

*SLC20A2* (solute carrier family 20, member 2), the first causative gene described [5], encodes the transmembrane sodium-inorganic phosphate cotransporter PiT2, which may have a role in phosphate clearance from the cerebrospinal fluid [6].

Several missense, nonsense mutations, and deletions of this gene have been described, and they may account for up to 17% of PFBC cases regardless of family history [4].

We confirm here the pathogenic role of three different mutations in *SLC20A2* gene in three patients with PFBC presenting with different clinical phenotypes.

## Methods

### Genetic analysis

DNA of the patients was extracted from peripheral blood using standard procedure. NGS analysis of case #2 was performed using Illumina’s TruSight ONE Sequencing Panel (tsONE, 4813 genes; Illumina, San Diego, CA, USA) with the MiSeq instrument, according to the manufacturer’s instructions. The instrument produced 2 × 150-bp read length based on 100× mean coverage of targeted content. The bioinformatics pipeline, used by MiSeq Reporter software, is based on tools for read mapping (Burrows-Wheeler Aligner—BWA), removal of duplicates followed by realignment (GATK software), base calling (SAMtools) in order to obtain a Variant Call Format (VCF) file. The variant annotation was performed with wAnnovar tool (http://wannovar.wglab.org/) using hg19 genome assembly (https://www.ncbi.nlm.nih.gov/assembly/GCF_000001405.13/). Relevant DNA sequence variants were confirmed by Sanger sequencing using standard protocols and using an automated 3130 XL DNA analyzer (Applied Biosystems, Foster City, CA, USA).

For case #3, the p.Val230Cysfs*28 variant was identified using a customized Movement Disorders gene panel as described in Reale C. et al. 2018 [7].

For cases of family #1, we confirmed the presence of the already reported c.290-8 A>G mutation [4], by Sanger sequencing.

### Characterization of *SLC20A2* expression for c.290-8 A>G mutation

Total RNA was extracted from peripheral blood mononuclear cells (PBMCs) using the RNeasy® Plus Mini Kit (Cat No./ID: 74134 QIAGEN) according to the manufacturer’s recommendation. Total RNA was used to produce cDNA using the GoScript™ Reverse Transcriptase (Cat# A5003, Promega). SLC20A2 cDNA was amplified using the following primers: forward 5′-CCGTGTTACTAGGCGCCAAAGTAG-3′, reverse: 5′-GCTCCTGTGTACATGATGGAAAA-3′. To amplify the specific intron-retention splicing isoform (transcript 3), PCR was performed on cDNA derived from patients with the c.290-8 A>G variant and healthy controls, using primers forward 5′-GGAAGTTAGTGCCATGGTTGTC-3′ and reverse: 5′-GTGCTCCTGTGTACATGATGG-3′. Glyceraldehyde-3-phosphate dehydrogenase (GAPDH) was used as housekeeping gene. The PCR products were verified by 2% agarose gel electrophoresis. All the RT-PCR product isoforms were isolated from gel using GeneJET PCR Purification Kit (Cat# K0702, Thermo Fisher Scientific) and analyzed by Sanger sequencing (BigDye Terminator Cycle Sequencing Kit version 3.1 (Applied Biosystems)) on an automated 3130 XL DNA analyzer (Applied Biosystems, Foster City, CA, USA).

## Results

### Case reports

#### Family 1

**Fig. 1:**
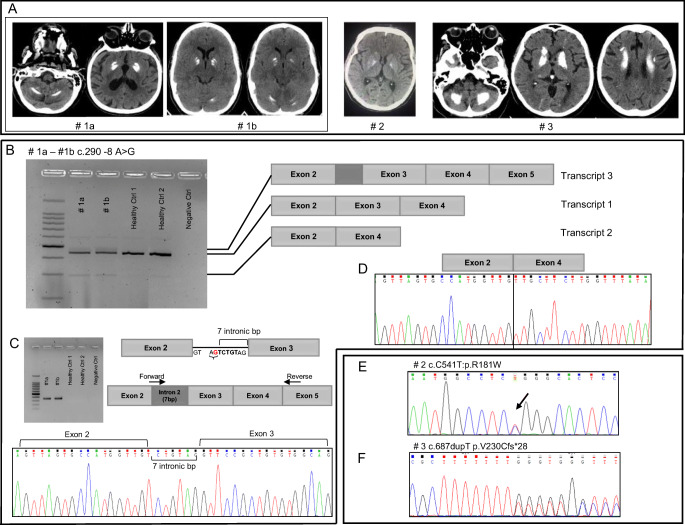
**a** Cerebral CT scans showing the localization of the brain calcifications in the described cases. **b** RT-PCR analysis on 2% agarose gel electrophoresis performed using RNA derived from PBMCs of the c.290-8 A>G mutation carriers (#1a, #1b) and healthy controls and schematic representation of the alternative splicing mechanisms involving *SLC20A2* (exons 2–4). The lines indicate the three different transcripts detected in the analyzed samples. **c** RT-PCR analysis on 2% agarose gel electrophoresis performed using RNA from PBMCs patients with c.290-8 A>G (#I.1, #I.2) and healthy controls using primers to amplify specifically the intron 2 retained isoform (transcript 3). The position of the new acceptor site and the primers used to amplify the aberrant isoform are graphically indicated. **d** Electropherogram relative to transcript 2 isoform showing exon 3 skipping. **e** Electropherogram relative to exon 5 of *SLC20A2* of the case #2 showing the c.541C>T p.R181W (NM_001257180) heterozygous missense mutation. **f** Electropherogram relative to exon 6 of *SLC20A2* gene in case #3 showing the c.687dupT p.V230Cfs*28 (NM_001257180) heterozygous mutation

The index patient of this family was previously described by Ramos et al. (IT-PFBC-1) [4]. She is a 74-year-old woman (Fig. 1a, #1a) which was referred to our Movement Disorder Center for a rest tremor started at the age of 65. She was diagnosed with Parkinson’s disease (PD) and started with pramipexole, rasagiline, and subsequently with levodopa with a good control of motor symptoms. During the workup evaluations, she performed a cerebral computerized tomography (CT) scan, revealing the presence of calcifications involving the putamen, caudate, and dentate nuclei bilaterally (total calcification score TCS = 14) [8]. No impairment in calcium metabolism was detected. Accordingly, a diagnosis of PFBC was made and genetic test for *SLC20A2* mutations was performed revealing the presence of an intronic variant c.290-8 A>G (NM_001257181.1) in heterozygous status (described as case ID: #IT-PFBC-1, [4]). The patient did not have a positive family history of neurological/psychiatric disorders.

Her 45-year-old daughter was recently referred to our Movement Disorder Center because of a mild subjective hypokinesia but without evidence of extrapyramidal signs. A CT scan was performed on her which showed the presence of bilateral calcifications in the basal ganglia and dentate nuclei that were less extended as compared to the mother (TCS = 10) (Fig. 1a, #1b). The genetic test performed by Sanger sequencing revealed the presence of the same *SLC20A2* intronic mutation detected in her mother. As this mutation was previously reported in a PFBC patient to induce partial intron retention [9], an *SLC20A2* expression analysis in PBMCs, of the proband (#1a), her asymptomatic daughter (#1b), and two healthy controls was performed to analyze SLC20A2 aberrant splicing isoforms.

Four *SLC20A2* PCR amplification products have been detected (Fig. 1b). A main transcript (transcript 1) was detected in both the mutation carriers and the two controls, with similar expression. An additional isoform (transcript 2) was found in both patients and controls albeit with higher expression in patients compared to controls (Fig. 1b). Direct sequencing of transcript 2 revealed the presence of exon 3 skipping isoform (Fig. 1d). Transcript 2 predicts an inframe loss of 47 amino acids (p.Gly97_Val145del) encoded by exon 3 nucleotides. A third isoform was found only in the two patients (transcript 3). To analyze the nature of transcript 3, we performed a PCR amplification using primers specific for the in silico predicted intron 2-retention isoform (Fig. 1c). The aberrant isoform was detected only in the mutation carriers and was absent in the two healthy controls (Fig. 1c). Sanger sequencing of this PCR product confirmed the presence of the last seven intron-2 nucleotides according to the use of the predicted new acceptor site in transcript 3 (Fig. 1c). Transcript 3 is predicted to lead a shorter protein (p.Gly97Valfs*162) due to a frameshift with introduction of 162 novel amino acids. The highest band (about 600 bp) detected in both patients and controls represents a non-specific amplification product. All the experiments were repeated twice (data not shown).

#### Family 2

A 60-year-old man (Fig. 1a, #2) was referred to our Tertiary ALS Centre for a 2-year slowly progressive history of dizziness; bilateral muscle cramps in the neck, back, and legs; and mild dysarthria and dysphagia. Medical history included blood hypertension and occupational hearing loss.

A non-defined neurodegenerative disorder was reported in his mother, with similar clinical features. Her medical record at age of 50 years reported limb rigidity, hand tremor, buccofacial dyskinesia, and severe dysarthria. She was diagnosed with an atypical Parkinsonism.

On first neurological examination, our patient showed a mild dysarthric speech, an ataxic gait with anteflexed head, mild generalized chorea, and a dystonic posture of the right hand. The neuropsychological assessment revealed severe behavioral and executive deficits. He underwent brain magnetic resonance imaging (MRI), which showed frontal bilateral atrophy and mild bilateral hypointensity in the basal ganglia in T2 sequences. Hematological screening (neoplastic, metabolic, and infection markers) was unremarkable. The needle electromyography (EMG) showed a severe and chronic neurogenic involvement and diffuse signs of active denervation (mainly with monomorphic fasciculations) in the cervical and lumbar regions, significant for the lower motor neuron involvement. The DAT-Scan and 18F-FDG-PET showed no significant abnormalities. After 6 months from the previous evaluation, the EMG findings were unchanged. In order to better investigate the basal ganglia MRI hypointensity, a cerebral computerized tomography scan was done, with evidence of bilateral calcifications in the basal ganglia (TCS = 6).

We followed the patient roughly for 10 years from symptom onset, and we observed a slowly progressive worsening of extrapyramidal, cognitive/behavioral, and MND symptoms. Nine years after the onset, he developed severe dysphagia and severe respiratory complications with hypercapnic hypoxemic respiratory failure. He underwent tracheotomy and percutaneous endoscopic gastrostomy (PEG) placement.

We performed NGS-based targeted resequencing analysis (Clinical Exome Illumina) in family 2 proband (#2). No mutation was detected in the known main ALS and PD-related genes available in the clinical exome panel. Mutational analysis revealed the presence of a missense heterozygous variant, c.541C>T (NM_001257180) p.R181W, located in exon 5 of *SLC20A2* gene, which was confirmed by Sanger sequencing (Fig. 1e). The variant was present in the single-nucleotide polymorphism database (dbSNP) (rs1211274033), and its frequency was extremely low (4.0e-0^6^ GnomAD public database, https://gnomad.broadinstitute.org).

The missense variant in *SLC20A2* is localized in one of the cytosolic domains of the PiT2 protein and is predicted to be damaging by 10 out of 11 different in silico functional prediction tools (data not shown). Since the mutation was found in other two patients showing cerebral calcifications [10, 11], it can be classified as “likely pathogenic” according to the ACMG guidelines [12].

#### Family 3

An 82-year-old woman (Fig. 1a, #3) was referred to our clinic because of a minor ischemic stroke. She had already been diagnosed with a cognitive impairment with a score of 11 at MOCA and 20.7 at MMSE and was on Donepezil. Her family history was unremarkable. Neurological examination was normal except for temporal and spatial disorientation.

A cerebral CT scan showed diffuse brain calcifications involving cerebellar dentate nuclei and basal ganglia (TCS = 26). No impairment in calcium metabolism was detected. A customed NGS panel for PFBC was performed as previously described [7]. The analysis revealed a heterozygous variant c.687insT in exon 6 of SLC20A2 gene which predicts the introduction of a premature stop codon (p.Val230Cysfs*28) (Fig. 1f). This variant is not present in the GnomAD public database (https://gnomad.broadinstitute.org) and is classified as “likely pathogenic” according to the ACMG/AMP guidelines [12]. No other family member performed a CT brain scan or genetic test.

## Discussion

In this paper, we report the phenotypic variability of mutations in *SLC20A2* gene found in three patients belonging to different families diagnosed with PFBC. In fact, while the proband of family 1 (#1A) presented as a Parkinson’s disease phenocopy, the proband of family 2 (#2) had an “atypical” presentation with a combination of motor neuron dysfunction and executive-behavioral impairment, and proband of family 3 (#3) presented with an isolated cognitive impairment.

The association of MND with cognitive and behavior impairment and primary brain calcifications represents a very rare condition. Based on the clinical, biochemical, and radiological findings, a diagnosis of MND and PFBC was made in our patient. Only another case of PFBC with MND was previously reported by Eleopra and colleagues [10]. Similarly to our patient, he showed an association of MND and cognitive impairment with a long course, and the CT scan revealed diffuse cerebral and cerebellar calcifications. No genetic test was performed.

ALS is recognized to be part of a continuum that includes other nosological conditions of the central nervous system (CNS). In particular, several studies suggest substantial overlap between FTD and MND syndrome with respect to clinical and pathological features and genotype [13]. In these cases, the most frequent mutations are detected in genes such as *C9ORF72*, *FUS*, and *TARDBP* [14]. Accordingly, in our patient, mutations in *HTT* and MND/FTD known causative genes have been excluded. The p.R181W missense mutation carried by the proband of family 2 (#II) is a rare *SLC20A2* variant (4.0e-06 in the GnomAD database). The R181 residue lies in one of the cytosolic domains of the *SLC20A2* transmembrane protein, where several other PFBC-associated *SLC20A2* variants have been described [15]. In addition, almost all (10 out of 11) of the in silico prediction tools suggest a pathogenic consequence of this amino acidic substitution. Unfortunately, segregation analysis was not possible in this family as no other family members performed a brain CT scan and genetic testing. The p.R181W variant has been previously reported in 2 PFBC patients with different clinical presentations: a subject presenting with progressive myoclonus, associated to neuropsychiatric symptoms, cramps, and fasciculations (in the absence of significant EMG findings) [11] and a patient showing progressive involuntary movements, neuropathic pain, and chronic headache with a positive family history [4]. In both cases, the possible pathogenicity was only hypothesized. The peculiarity of our patient relies on the association of lower motor neuron sufferance (confirmed by EMG) and cerebral calcification due to mutation in *SLC20A2*. Although we cannot exclude the co-occurrence of two different neurological diseases in the same patient, the presence of MN involvement and cerebral calcification may widen the clinical spectrum of PFBC patients. All these in silico data, together with the description of this mutation in other two PFBC patients, strongly support a causative role of the p.R181W mutation in PFBC. Despite haploinsufficiency, due to the presence of loss of function mutations, being considered the molecular mechanism underlying the majority of *SLC20A2*-related PFBC cases [15], previous studies demonstrated also for some *SLC20A2* nonsense and missense mutations a significant reduction of SLC20A2 expression in the blood samples [16–18].

The first case (#1a) carried the c.290-8 A>G intronic variant in *SLC20A2* gene. The same mutation was identified in her daughter presenting basal ganglia and cerebellar calcifications (Fig. 1a). Our results showed that, besides the canonical transcript (namely, transcript 1), two other aberrant transcripts, due to the intronic nucleotide substitution, were generated with different impact on SLC20A2 protein.

Transcript 3 was detected exclusively in mutation carriers and is predicted to introduce after the glycine 97, 162 novel amino acids before the stop codon, as previously described by Chen et al. [9]. Transcript 2 is the result of exon 3 skipping, and the expression of this shorter isoform seems to be higher in mutation carriers than in controls suggesting a direct consequence of the mutation (Fig. 1b). Exon 3 skipping is predicted to cause an inframe deletion of 47 amino acid residues belonging to two different SLC20A2 transmembrane domains. The presence of aberrant transcripts associated with the c.290-8 A>G intronic variant and its segregation in two family members with symptoms attributable to PFBC strongly support the causative role of this mutation in PFBC.

The third PFBC case (#3) carried a novel *SLC20A2* p.Val230Cysfs*28 mutation. Also, this mutation causes a frameshift and a premature SLC20A2 stop codon. Unlike the phenotype showed by patient 2, patient 3 presented a “more common” PFBC phenotype, although both the patients carried a truncating *SLC20A2* mutation. Further studies of patients carrying similar pathogenic variants, but different disease course, will be necessary to shed light about other genetic/epigenetic or environmental factors which could act as modifier of disease progression.

In conclusion, our cases widen the genetic spectrum of PFBC caused by *SLC20A2* mutations and indicate a pathological role of three novel *SL20A2* variants. These data could be helpful for the interpretation of genetic data from future genetic screening of other PFBC patients and for the relative genetic counseling.
